# Primary Dysmenorrhea Induced Using Diethylstilbestrol and Oxytocin Induces Impaired Uterine Reactivity in Virgin Female Wistar Rats

**DOI:** 10.3390/ph18081191

**Published:** 2025-08-13

**Authors:** Francisco Fernandes Lacerda-Júnior, Petruska Pessoa da Silva Souza, Paula Benvindo Ferreira, Anderson Fellyp Avelino Diniz, Bárbara Cavalcanti Barros, Maria da Conceição Correia Silva, Adriano Francisco Alves, Alexandre Sérgio Silva, Bagnólia Araújo da Silva

**Affiliations:** 1 Postgraduate Program in Natural and Synthetic Products Bioactives, Health Sciences Center, Federal University of Paraíba, João Pessoa 58051-900, Brazil; paulabenvindo92@hotmail.com (P.B.F.); barbaracavalcanti@ltf.ufpb.br (B.C.B.); adrianofalves@gmail.com (A.F.A.); bagnolia@ltf.ufpb.br (B.A.d.S.); 2 Health Sciences Center, Federal University of Paraíba, João Pessoa 58051-900, Brazil; petruskapessoa@gmail.com; 3 Postdoctoral National Council for Scientific and Technological Development, Functional Pharmacology Laboratory, Federal University of Paraíba, João Pessoa 58051-900, Brazil; andersonfellyp@gmail.com; 4 Postdoctoral National Council for Scientific and Technological Development, Foundation for the Support of Science and Technology of Pernambuco, Recife 50740-465, Brazil; ceicafarma@gmail.com; 5 Department of Biomedical Sciences, Health Sciences Center, Federal University of Paraíba, João Pessoa 58051-900, Brazil; 6 Department of Physical Education, Health Sciences Center, Federal University of Paraíba, João Pessoa 58051-900, Brazil; alexandresergiosilva@yahoo.com.br; 7 Functional Pharmacology Laboratory, Pharmaceutical Sciences Department, Health Sciences Center, Federal University of Paraíba, João Pessoa 58051-900, Brazil

**Keywords:** female rats, oxytocin, diethylstilbestrol

## Abstract

**Background/Objectives:** Primary dysmenorrhea (DysP) is a prevalent gynecological condition characterized by painful uterine contractions. However, the underlying mechanism of action of dysmenorrhea has not been fully elucidated. This study aimed to standardize an animal model of dysmenorrhea using diethylstilbestrol and oxytocin to mimic pathophysiological mechanisms in female Wistar rats. Methods: For the induction of dysmenorrhea, diethylstilbestrol (s.c.) and oxytocin (i.p.) were used. Results: The model effectively reproduced hypercontractility and impaired uterine relaxation. The in vivo evaluations demonstrated increased pain responses (DysP group = 119 ± 6.9; control group CG = 3.0 ± 1.0), which were partially attenuated by standard medications (scopolamine/dipyrone and ibuprofen). In vitro assays revealed greater contractile reactivity when compared to that in the control group, in the DysP group, using oxytocin (pEC_50_ = 3.6 ± 0.2 and E_max_ = 145.1 ± 8.7; CG (pEC_50_ = 3.1 ± 0.1 and E_max_ = 100%); KCl (DysP pEC_50_ = 2.2 ± 0.1 and E_max_ = 164 ± 8.0); CG (pEC_50_ = 1.8 ± 0.1) and PGF_2α_ (DysP pEC_50_ = 7.4 ± 0.2 and E_max_ = 127.3 ± 15.6); CG (pEC_50_ = 6.2 ± 0.1)), while the relaxation responses to isoprenaline and nifedipine were decreased compared to those in the CG. The model promoted an imbalance in oxidative stress by increasing malondialdehyde (MDA) levels and reducing the total antioxidant capacity (TAC) in the uterine tissue. Conclusions: These findings suggest that the new virgin rat model is capable of replicating key aspects of the clinical features of DysP in humans and offers a valuable tool for studying its pathogenetic mechanisms and testing potential therapeutic agents.

## 1. Introduction

Primary dysmenorrhea (DysP), popularly known as menstrual cramps, is a common gynecological disorder that affects between 45% and 95% of women of reproductive age; it is classified as primary dysmenorrhea and secondary dysmenorrhea according to its etiology. DysP is caused by uterine hypercontraction without any identifiable pelvic lesions, while secondary dysmenorrhea is brought about by gynecological disorders with pelvic organic lesions. DysP is characterized by menstrual pain without organic lesions in the pelvic cavity and spastic pain and distension in the lower abdominal region [1,2,3]. It can often be accompanied by other biological symptoms such as exhaustion, sweating, headaches, nausea, vomiting, and diarrhea; DysP represents the main cause of impediments to young women carrying out their daily activities normally, such as attending school or going to work [4,5,6].

Despite it being an impactful, frequent, and debilitating public health problem, it is still an underreported and undertreated disease, whose underreporting has been attributed to the fact that the pathophysiological mechanisms of DysP are not yet fully understood [7,8]. It is believed that the etiology of DysP is mainly related to the production of prostaglandins, which generate uterine contractions, resulting in ischemia and nerve sensitivity throughout the uterus [9,10].

In this context, one of the main inflammatory mechanisms involved in the pathophysiology of DysP occurs due to the exfoliation of endometrial epithelial cells, decreased progesterone levels, and the consequent activation of metalloproteinases (1, 2, and 3), which lead to the degradation of the endometrial extracellular matrix, releasing membrane phospholipids, substrates for the action of phospholipase A_2_ (PLA_2_), which converts them into arachidonic acid, which is subsequently converted by cyclooxygenases -1 and -2 into prostaglandins (PGs), prostacyclin, and thromboxane, with PGF_2α_ being one of the most important agents in increasing uterine contractility during menstruation [11,12,13,14].

Thus, the hyperproduction of prostaglandins, particularly PGF_2α_, is attributed to uterine colic, given that these are responsible for promoting an increase in the tone and amplitude of contractions, developing abnormal contractile activity in the uterus, and narrowing the blood vessels, leading to uterine ischemia and hypoxia and increased sensitivity in nerve endings [15,16,17]. Hypoxia and ischemia-re perfusion conditions in the uterus trigger an increase in radical species and causes a decrease in the activity of antioxidant enzymes, in turn resulting in increased oxidative stress, leading to changes in uterine signaling and hindering myometrial relaxation [18].

A variety of treatments have been made available for DysP, aiming to reduce symptoms and increase the quality of life in women affected by this disorder. Non-pharmacological measures mainly include alternative practices such as acupuncture [19], aromatherapy [20], and physical exercise [21]. Among pharmacological measures, non-steroidal anti-inflammatory drugs (NSAIDs), calcium channel blockers, antispasmodics, and combined hormonal contraceptives stand out [17,22]. However, despite them being described as the first-choice treatment, numerous undesirable effects are evidenced, such as gastrointestinal and liver disorders [23,24].

Given this context, this study was conducted to develop the standardization of a model of primary dysmenorrhea induced using diethylstilbestrol and oxytocin in female Wistar rats capable of mimicking the characteristics of hypercontractility and decreased relaxation in the uterine smooth muscle, similar to what occurs in human primary dysmenorrhea, thus providing a useful tool for the study and greater understanding of the mechanisms of action involved in the pathophysiology of this disorder, as well as determining more effective pharmacological approaches to DysP. We present an in vivo and in vitro naïve female mouse model that can be used to study DysP and for future clinical therapeutic evaluations in order to provide a reference for the selection of murine dysmenorrhea models and future studies on the pathophysiological mechanisms of DysP. The establishment of this model can be used to screen for more effective pharmacological treatments with fewer side effects.

## 2. Results

### 2.1. Writhing Scores of the Experimental Model of Primary Dysmenorrhea in Female Wistar Rats

**Figure 1:**
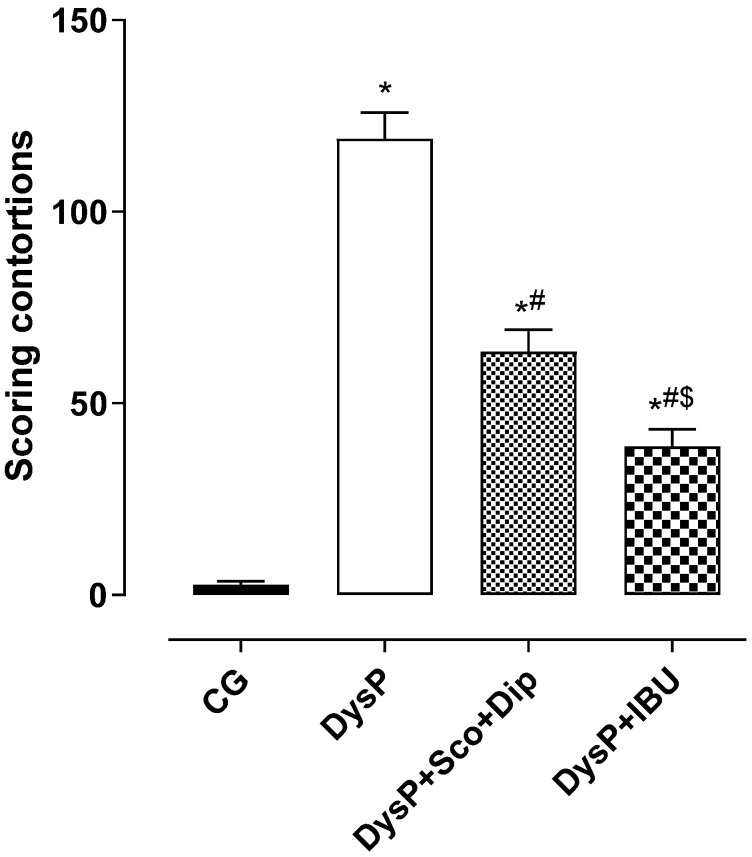
Scores regarding the contortions of the groups, CG, DysP, DysP + Sco + Dip, and DysP + IBU, in isolated rat uteri. Symbols and vertical bars represent the mean and s.e.m., respectively (*n* = 10). We used one-way ANOVA followed by Tukey’s post-test. * *p* < 0.05 (CG vs. DysP); ^#^ *p* < 0.05 (DysP vs. DysP + Sco + Dip; DysP + IBU); and ^$^ *p* < 0.05 (DysP + IBU vs. DysP + Sco + Dip). CG = control group; DysP = primary dysmenorrhea group; DysP + Sco + Dip = primary dysmenorrhea + scopolamine with dipyrone (0.46/23 mg/kg) group; DysP + IBU = primary dysmenorrhea + ibuprofen (50 mg/kg) group.

When analyzing the writhing scores induced by the experimental method of primary dysmenorrhea in female Wistar rats, we observed that the score of the DysP group (119 ± 6.9) was increased when compared to the control group (3.0 ± 1.0). The groups treated with the standard drugs, scopolamine and dipyrone (DysP + Sco + Dip) (64.0 ± 5.8) and ibuprofen (DysP + IBU) (39.0 ± 4.5), presented a decrease in the writhing score when compared to the DysP group (Figure 1).

### 2.2. Evaluation of the Effects of Primary Dysmenorrhea on the Histomorphometric Parameters of the Uterus

**Figure 2:**
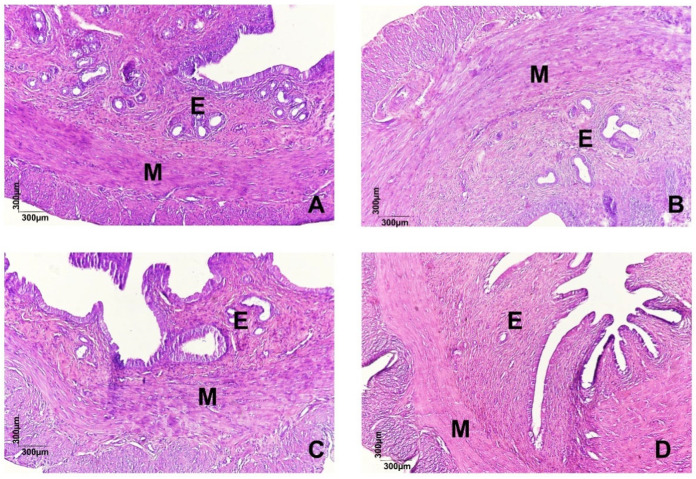
Effects of primary dysmenorrhea on uterine morphology (**A**). A photomicrograph of the uterus (**A**) of a rat in the CG (**A**), DysP (**B**), DysP + Sco + Dip (**C**) and DysP + IBU (**D**). The endometrium (E) and myometrium (M) can be seen. CG = control group; DysP = primary dysmenorrhea group; DysP + Sco + Dip = primary dysmenorrhea + scopolamine with dipyrone (0.46/23 mg/kg) group; DysP + IBU = primary dysmenorrhea + ibuprofen (50 mg/kg) group.

In the histological section of the uteri (Figure 2A) stained with hematoxylin and eosin, parts of the organs are shown, highlighting the endometrium (E) and myometrium (M). In the animals of the CG (A), the histologically preserved endometrium, circular glandular conformations, and the absence of morphological lesions are observed. Unlike in the DysP, DysP + Sco + Dip, and DysP + IBU groups, disorganized endometrial glands and damage to the endometrial tissue (E) are observed, in addition to the thickening of the myometrial layer (M) when compared to the CG.

**Figure 3:**
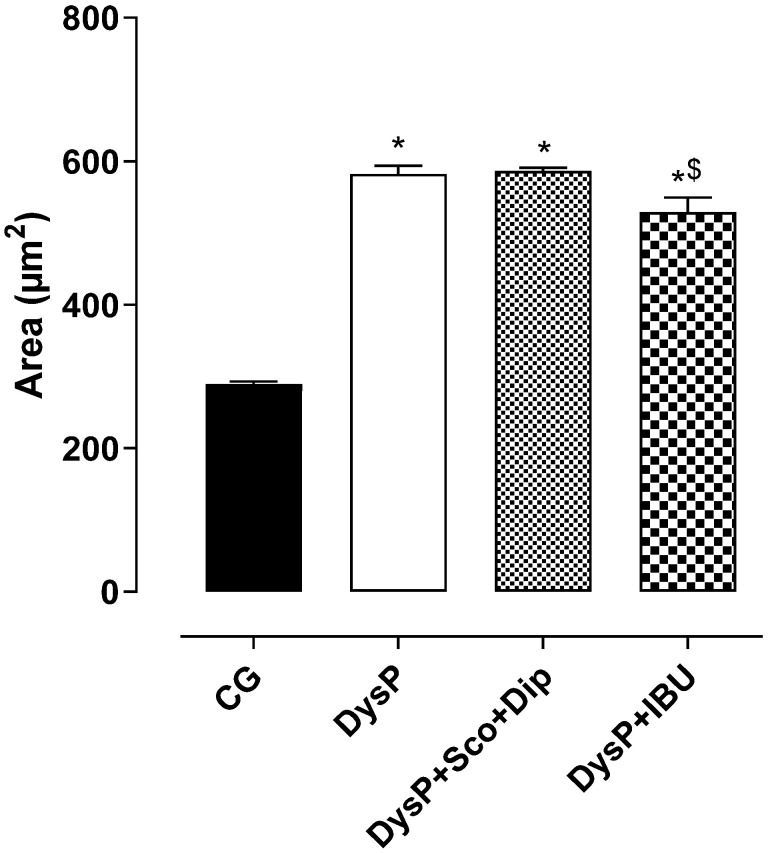
Effects of primary dysmenorrhea on the muscular area in the rat uterus in the CG and DysP, DysP + Sco + Dip, and DysP + IBU groups. Symbols and vertical bars represent the mean and s.e.m., respectively (*n* = 10). We used one-way ANOVA followed by Tukey’s post-test. * *p* < 0.05 (CG vs. DysP, DysP + Sco + Dip, and DysP + IBU); ^$^ *p* < 0.05 (DysP + IBU vs. DysP + Sco + Dip). CG = control group; DysP = primary dysmenorrhea group; DysP + Sco + Dip = primary dysmenorrhea + scopolamine with dipyrone (0.46/23 mg/kg) group; DysP + IBU = primary dysmenorrhea + ibuprofen (50 mg/kg) group.

When analyzing the myometrial layer (M), the morphometric data indicate that when compared to female rats in the CG (289.2 ± 3.8 µm^2^), the myometrial layer of the DysP group (582 ± 11.5) increased. However, the groups treated with the standard drugs, DysP + Sco + Dip (586.2 ± 4.6 µm^2^) and DysP + IBU (528.8 ± 20.2 µm^2^), did not reverse the increase when compared to the DysP group (Figure 3).

### 2.3. Evaluation of the Effects of Primary Dysmenorrhea on Oxytocin- or PGF_2α_-Induced Contractile Reactivity in the Uterus of Female Wistar Rats

**Figure 4:**
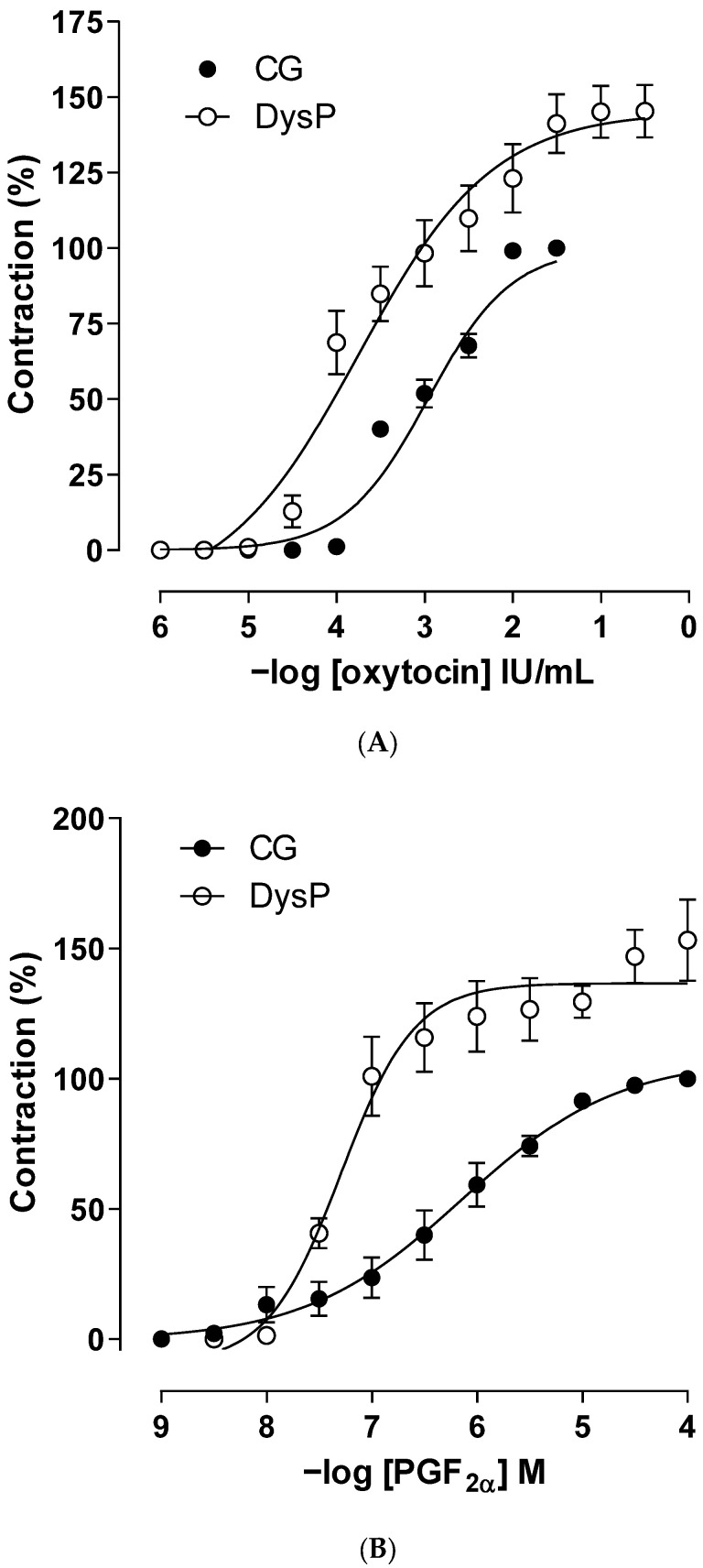
Cumulative concentration–response curves for oxytocin (**A**) or PGF_2α_ (**B**) in the CG (●) and DysPgroup (◌) in isolated rat uteri. Symbols and vertical bars represent the mean and s.e.m., respectively (*n* = 10). A *t*-test was used; DysP = primary dysmenorrhea group and CG = control group.

In the results obtained, it is observed that the cumulative oxytocin contraction curve of the DysP group is shifted to the left with an increase in the contractile power (pEC_50_ = 3.6 ± 0.2) and maximum effect (E_max_ = 145.1 ± 8.7) of the agonist when compared to the CG (pEC_50_ = 3.1 ± 0.1 and E_max_ = 100%), respectively (Figure 4A and Table 1).

When evaluating the changes in reactivity to the PGF_2α_ agonist, it is observed that the curve of the DysP group shows a shift to the left, increasing the potency of the agonist (pEC_50_ = 7.4 ± 0.2) when compared to the CG (pEC_50_ = 6.2 ± 0.1). Similarly, an increase in the maximum effect of PGF_2α_ was observed in the DysP group (E_max_ = 127.3 ± 15.6) when compared to the CG (E_max_ = 100%) (Figure 4B and Table 1).

### 2.4. Evaluation of the Effects of Primary Dysmenorrhea on the Electromechanical Coupling of Kcl-Induced Contraction in the Uterus of Female Wistar Rats

**Figure 5:**
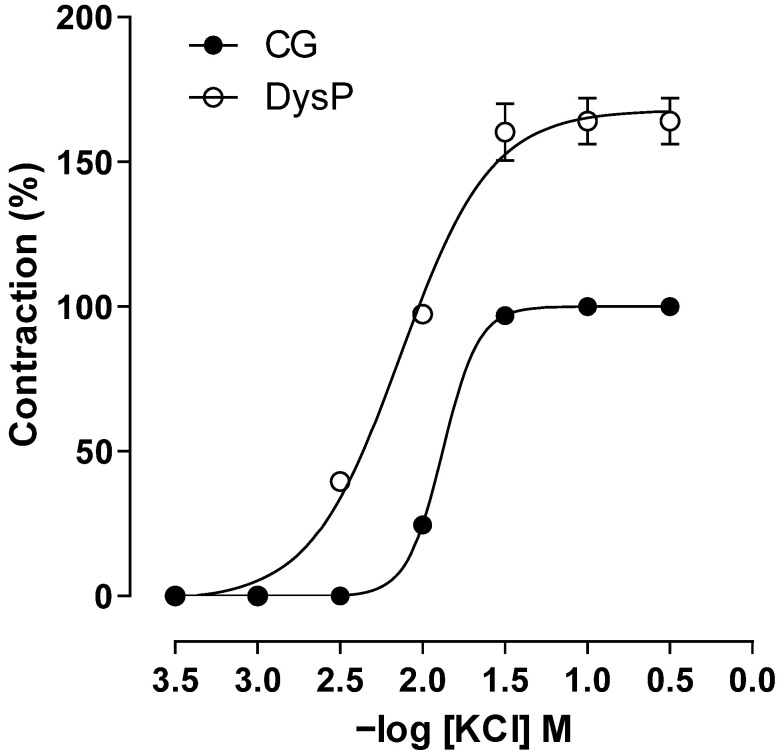
Cumulative concentration–response curves forKClin the CG (●) and DysPgroup (◌) in isolated rat uteri. Symbols and vertical bars represent the mean and s.e.m., respectively (*n* = 10). We used a *t*-test; DysP = primary dysmenorrhea group and CG = control group.

By evaluating the changes in primary dysmenorrhea in electromechanical coupling induced using KCl, it is observed that in the DysP group, the cumulative contraction curve induced using KClis shifted to the left, with an increase in the potency of the contractile agent (pEC_50_ = 2.2 ± 0.1) when compared to the CG (pEC_50_ = 1.8 ± 0.1); similarly, an increase in the maximum effect of KCl was observed in the DysP group when compared to the CG (E_max_ = 164 ± 8.0 and 100.0%, respectively) (Figure 5).

### 2.5. Evaluation of the Effects of Primary Dysmenorrhea on the Cumulative Concentration–Response Curves of Relaxation Induced Using Isoprenaline in Isolated CG (●) and Dysp (◌) Rat UteriPre-Contracted with Oxytocin

**Figure 6:**
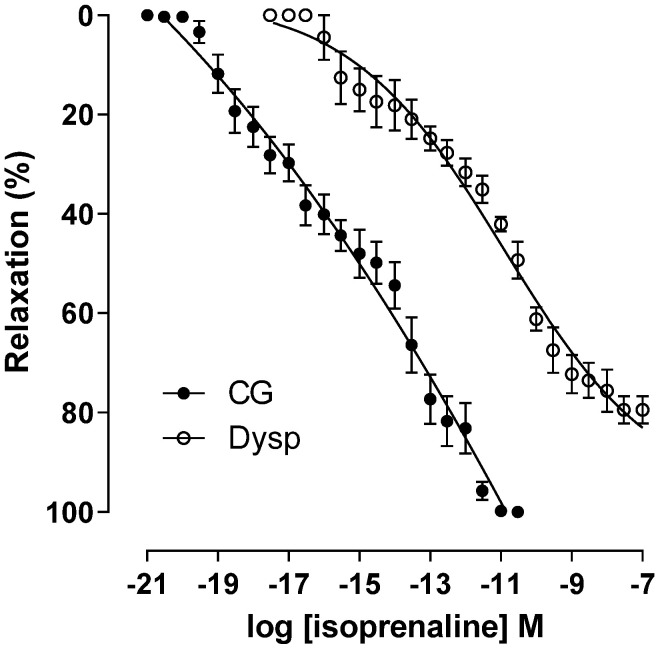
Effects of primary dysmenorrhea on the cumulative concentration–response curves of relaxation induced using isoprenaline in isolated CG (●) and DysP (◌) rat uteri pre-contracted with oxytocin. Symbols and vertical bars represent the mean and s.e.m., respectively (*n* = 10). A *t*-test was used; DysP = primary dysmenorrhea group and CG = control group.

In the DysP group, the cumulative relaxation curve with isoprenaline shows a shift to the right, with a decrease in agonist potency (pEC_50_ = 11.0 ± 0.2) when compared to the CG (pEC_50_ = 15.1 ± 0.2). A decrease in the maximum effect of isoprenaline was also observed in the DysP group when compared to the CG (E_max_ = 79.4 ± 2.7 and 100%, respectively) (Figure 6).

### 2.6. Evaluation of the Effects of Primary Dysmenorrhea on the Pharmacomechanical Coupling of Nifedipine-Induced Relaxation in the Uterus of Female Wistar Rats

**Figure 7:**
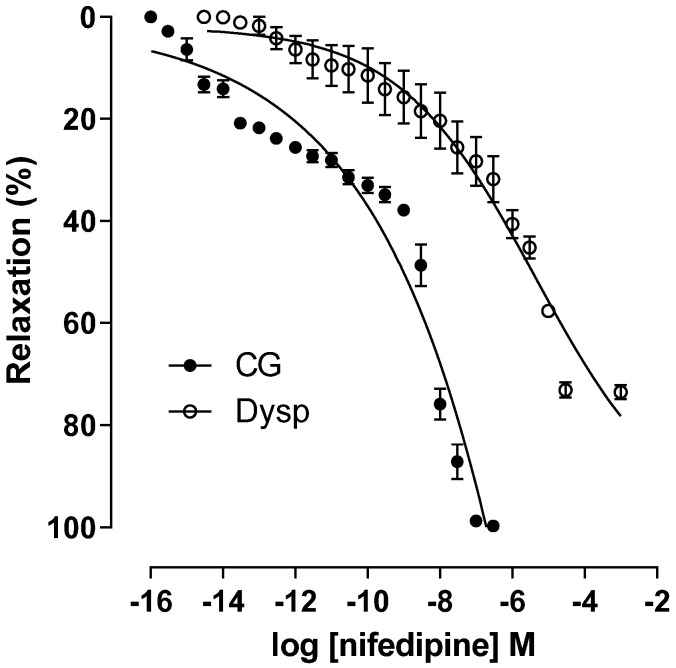
Effects of primary dysmenorrhea on the cumulative concentration–response curves of nifedipine-induced relaxation in isolated CG (●) and DysP (◌) rat uteri precontracted with KClin. Symbols and vertical bars represent the mean and s.e.m., respectively (*n* = 10). A *t*-test was used; DysP = primary dysmenorrhea group and CG = control group.

In the DysP group, the cumulative relaxation curve with nifedipine is shifted to the right with a decrease in the potency of the relaxing agent (pEC_50_ = 6.6 ± 0.1) when compared to the CG (pEC_50_ = 9.5 ± 0.1). In addition, there was a decrease in the maximum effect of nifedipine in the DysP group when compared to the CG (E_max_ = 73.5 ± 1.3 and 100 ± 0.1%, respectively) (Figure 7).

### 2.7. Evaluation of the Effects of Primary Dysmenorrhea onMDA Concentration in the Uterus of Female Wistar Rats

**Figure 8:**
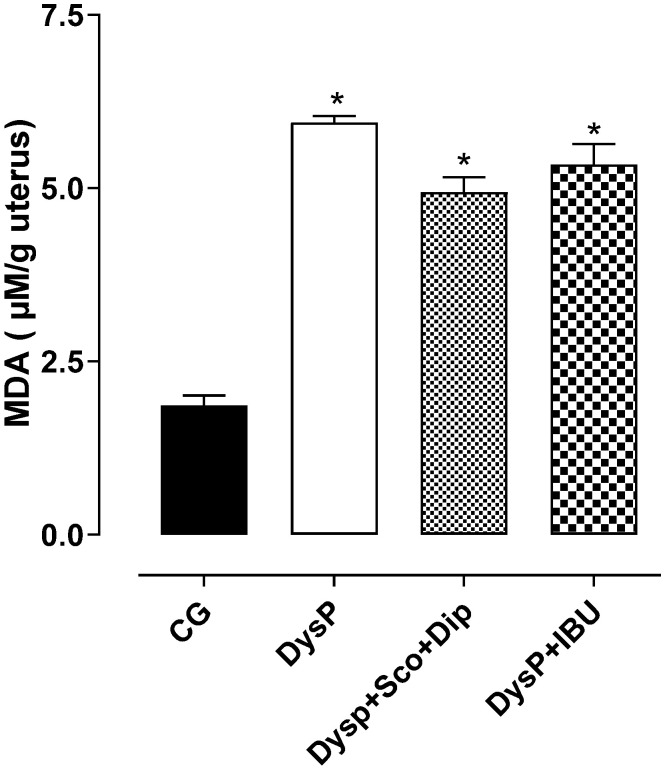
Effects of primary dysmenorrhea on malondialdehyde (MDA) levels in rat uteri in the CG and DysP, DysP + Sco + Dip, and DysP + IBU groups. Symbols and vertical bars represent the mean and s.e.m., respectively (*n* = 10). We used one-way ANOVA followed by Tukey’s post-test. * *p* < 0.05 (CG vs. DysP, DysP + IBU, and DysP + Sco + Dip). CG = control group; DysP = primary dysmenorrhea group; DysP + Sco + Dip = primary dysmenorrhea + scopolamine with dipyrone (0.46/23 mg/kg) group; DysP + IBU = primary dysmenorrhea + ibuprofen (50 mg/kg) group.

In the DysP group (6.0 ± 0.1%), an increase in MDA levels was observed in the uterus of female rats when compared to the CG (1.9 ± 0.2%); the same was observed in the DysP + IBU (5.3 ± 0.3%) and DysP + Sco + Dip (4.9 ± 0.2%) groups (Figure 8).

### 2.8. Evaluation of Effects of Primary Dysmenorrhea onTotal Antioxidant Capacity (TAC) in Rat Uterus

**Figure 9:**
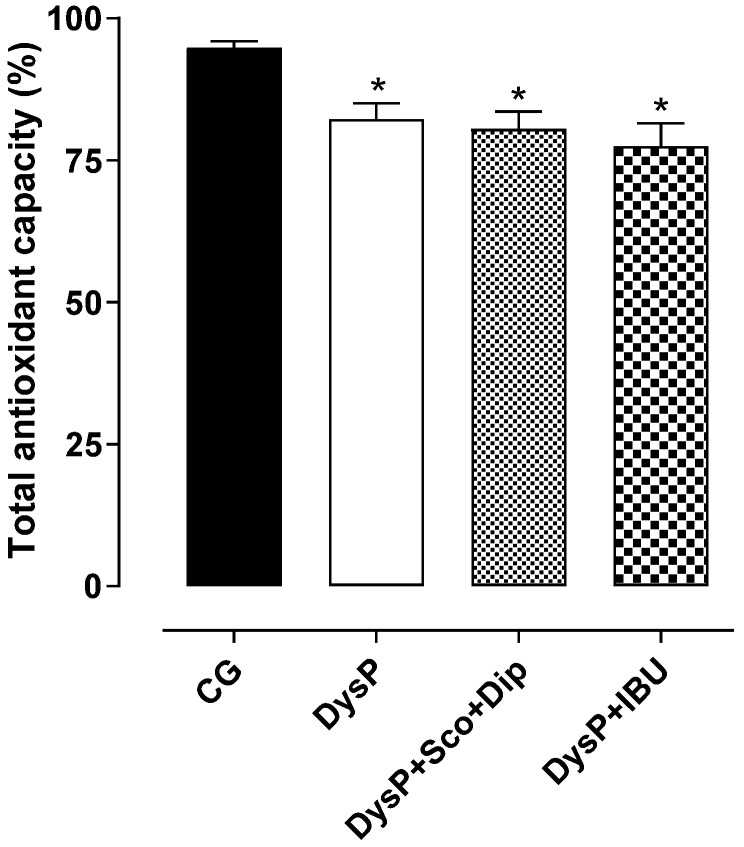
Effects of primary dysmenorrhea on the total antioxidant capacity (TAC) in the uterus of female rats in the CG andDysP, DysP + Sco + Dip, and DysP + IBU groups. Symbols and vertical bars represent the mean and s.e.m., respectively (*n* = 10). We used one-way ANOVA followed by Tukey’s post-test. * p < 0.05 (CG vs. DysP, DysP + Sco + Dip, and DysP + IBU). CG = control group; DysP = primary dysmenorrhea group; DysP + Sco + Dip = primary dysmenorrhea + scopolamine with dipyrone (0.46/23 mg/kg) group; DysP + IBU = primary dysmenorrhea + ibuprofen (50 mg/kg) group.

In the uterus of female rats submitted to the dysmenorrhea protocol, there was a decrease in the TAC (82.2 ± 2.9%) when compared to the control group (94.8 ± 1.2%). The standard drugs (DysP + Sco + Dip (80.5 ± 3.1%) and DysP + IBU (77.5 ± 4.0%)) did not prevent the decrease in the TAC promoted by DysP (Figure 9).

## 3. Discussion

In this work, an experimental model of primary dysmenorrhea was standardized using the administration of diethylstilbestrol and oxytocin. Their effects were evaluated in vivo, through the scoring of contortions, and in vitro, verifying changes in histomorphology and oxidative stress, in addition to the contractile and uterine relaxant reactivity of female Wistar rats.

Primary dysmenorrhea is characterized by increased uterine contraction, which triggers increased pelvic pain in women [25]. In this context, to evaluate the effectiveness of implementing this model, a behavioral study was carried out, evaluating writhing scores in female rats after the administration of estradiol and oxytocin. The results obtained showed an increase in the contortion score of the DysP group when compared to the healthy group, and when treated with scopolamine butyl bromide and dipyrone (DysP + Sco + Dip—0.46/23 mg/kg) or ibuprofen (DysP + IBU—50 mg/kg), the contortion scores were reduced in the DysP group. Thus, the use of first-choice drugs for the treatment of primary dysmenorrhea is based on their mechanisms of action, involving the use of non-steroidal anti-inflammatory drugs, such as ibuprofen and dipyrone, which are responsible for inhibiting cyclooxygenases, reducing the formation of contractile prostanoids such as PGF_2α_ and thromboxane A_2_, important mediators in the pathogenesis of DysP. Furthermore, the use of antispasmodics such as scopolamine, a muscarinic M3 antagonist, antagonizes the actions of acetylcholine in these receptors, decreasing cytosolic calcium levels resulting in the relaxation of uterine smooth muscle [3,26,27].

Thus, one of the parameters used to confirm the efficacy and development of primary dysmenorrhea in female rats and mice is through the evaluation of uterine histomorphological aspects, as observed in human DysP by the increase in the myometrial layer and damage to the endometrial layer [28]. In this study, the dysmenorrhea model promoted a rise in the myometrial layer and damage to the endometrial layer, confirmed by the disorganization of the endometrial glands. In addition, the groups treated with the standard drugs Esc + dysp and DysP + IBU did not reverse these changes. Thus, we demonstrated that the model evidenced in this study resembles the characteristics observed in human dysmenorrhea. However, treatment with standard drugs did not reverse these changes, ruling out the action of these drugs on the signaling pathways involved in cell proliferation and growth in the uterus [29].

The increase in uterine contractility during menstruation is a physiological process that facilitates the expulsion of endometrial tissue and, in women with primary dysmenorrhea, a dysregulation in this process is observed, causing the appearance of pelvic pain [29]. Uterine contraction can be regulated by the action of some hormones, such as oxytocin, whose mechanism of action plays a fundamental role in labor [30,31].

In this context, the primary dysmenorrhea induction protocol used in this study was based on the administration of diethylstilbestrol, an estradiol analog. Thus, the response to oxytocin was expected to be increased. In the female rats of the DysP group, a 1.82-fold potentiating of the effect of the contractile agonist was observed, as well as of the maximum effect, thus suggesting that the hormone may have increased the number of OT receptors and the affinity of oxytocin occupation to its pharmacological target. In contrast, the increase in the maximum effect was related to the increase in the expression or positive modulation of contractile proteins in the calcium sensitization pathway (Rho-ROCK) [32]. Furthermore, the activation of OT receptors can also result in the upregulation of PLA2 and COX-2, resulting in the increased production of PGs [25] such as PGF2α by endometrial cells [33,34].

Given these premises, contractions were induced with synthetic PGF_2α_. It was observed that the contraction curve of the DysP group was shifted 1.4 times to the left, with an increase in contractile power and efficacy, indicating that the pathophysiological changes induced by the dysmenorrhea model under study involved the positive modulation of the prostaglandin pathway, possibly due to the increased expression of FP receptors in the uterine myometrium and greater affinity of PGF_2α_ to its receptors.

In this context, the process of uterine smooth muscle contraction occurs through electromechanical and pharmacomechanical coupling. Uterine contraction is directly linked to an increase in cytosolic calcium concentration ([Ca^2+^]_c_) [35,36,37]. An estrogen-promoted upregulation of potassium channel expression was recently described, suggesting that this may be one of the steps by which there is an increase in uterine contractility in primary dysmenorrhea [38,39,40].

Thus, we decided to evaluate the participation of Ca_v_ channels, inducing cumulative concentration curves with KCl. It was observed that the curve of the DysP group was potentiated with a shift to the left of 1.8 times and greater efficacy with KCl, possibly indicating an increase in Ca_v_ expression or an increase in the influx of Ca^2+^ ions, involved in the pathophysiology of this disease.

The inhibition of Ca_v_ channels and decreased calcium ion influx is a mechanism involved in uterine relaxation, triggering a decrease in [Ca^2+^ ]_c_, which can occur through an electromechanical mechanism, leading to the repolarization or hyperpolarization of the membrane, or through a pharmacomechanical mechanism, from the activation of membrane receptors and inhibition of biochemical pathways that lead to the contraction or activation of relaxing pathways. Given this, uterine relaxation can be regulated by the activation of metabotropic receptors, such as in the adrenergic pathway (β_2_) [41,42].

Based on this, in cases of primary dysmenorrhea, it is suggested that β-adrenergic transduction is impaired due to decreased progesterone levels. Progesterone is responsible for regulating some steps in this pathway, increasing the expression of β_2_-adrenergic receptors and positively regulating the G_αs_ protein, resulting in greater coupling and activation in these receptors and adenylyl cyclase, resulting in uterine relaxation [11,43,44,45]. Thus, cumulative relaxation curves were obtained with isoprenaline, a β_2_ receptor agonist. We demonstrated that the relaxing response of the DysP group was hampered, confirmed by the 1.2-fold shift in the curve to the right and a decrease in relaxing potency and efficacy, thus evidencing a reduction in progesterone levels in these animals and/or the inhibition of steps involved in the β-adrenergic signaling pathway to promote the relaxation of the uterine myometrium.

Uterine relaxation in electromechanical coupling occurs, for example, by the opening of K^+^ channels, regulating the resting membrane potential and the excitability of smooth muscle cells and depolarization or hyperpolarization processes in the membrane. Recently, it was described that progesterone positively regulates the expression of K^+^ channels and estrogen does so for the expression of Ca_v_ in the uterine myometrium. Given the above, increased estradiol levels in the DysP setting can result in increased Ca_v_ expression and increased uterine contractility [40,46]. To confirm this hypothesis, nifedipine, an L-type Ca_v_ blocker, was used. We observed a shift in the nifedipine curve to the right, by approximately 1.1 times, with a decrease in the potency and relaxing efficacy of the blocker. These findings corroborate the result observed in contractile reactivity with the KCl agent.

Uterine hypercontractility promoted by increased Ca^2+^ ions in the uterus results in abnormal myometrial contractions and the vasoconstriction of the uterine arteries, developing ischemia and hypoxia phenomena, causing pelvic pain. Ischemia/hypoxia events induced during uterine contraction, due to decreased blood flow to the myometrium, can trigger the accumulation of free radicals (ROS). The increase in reactive species in the female reproductive system is responsible for the development of oxidative events, such as lipid peroxidation. Given this, we decided to evaluate whether lipid peroxidation could be involved in the DysP model under study.

We evidenced increased MDA levels in the uterus and ovaries in the DysP group, demonstrating the involvement of lipid peroxidation, caused by the increase in ROS in DysP, which in turn resulted in cellular damage and the activation of cellular processes involved in increased uterine contractility and inflammation, such as the release of contractile prostanoids (PG’s) through the activation of COXs and increased production of pro-inflammatory factors (NF-kB). These findings corroborate studies in which other DysP models have shown an increase in MDA levels in the female rat uterus [47,48].

Knowing that in DysP cases there is an increase in ROS production and the consequently suppression of antioxidant enzymes such as superoxide dismutase and catalase [49], we evaluated possible changes in the oxidative balance and total antioxidant capacity in the uterus. We observed that in the DysP group, there was a decrease in the uterine TAC, favoring oxidative imbalance in the female reproductive system, confirming the participation of hypoxia/ischemia events evidenced in the model under study in the generation of tissue ROS. These findings corroborate research that has shown that in inflammation conditions, such as DysP, there is a significant increase in the production of radical species due to the suppression of antioxidant systems in the female reproductive system [50].

Therefore, given these findings, we provide initial evidence that the DysP model used was effective in increasing contractile reactivity and decreasing uterine relaxant reactivity, being related to the increase in the myometrial layer and mechanisms that involvedthe increased expression of oxytocin receptors and prostaglandins and/or the decreased activation of β-adrenergic signaling, as well as possibly positively modulating the expression of L-type Ca_v_ or the influx of Ca^2+^ ions. Taking these results together, we demonstrate that the primary dysmenorrhea model of this study promoted oxidative imbalance in ROS, evidenced by the increase in MDA levels and decrease in catalase. Thus, it is demonstrated that the standardization of the model in the uterus of female Wistar rats can contribute to future studies of the signaling and pathophysiology of DysP and in the elucidation and discovery of new substances that may be candidates for drugs/medications in the prevention and/or treatment of primary dysmenorrhea. Although our study expands previous research in the field, one of the limitations is the lack of the verification of the normality of the distribution of the results using a more specific post hoc test (such as the Shapiro–Wilk test). Finally, the relationship between the histomorphometric parameters needs to be investigated further to understand the potential deleterious effects promoted by DysP in the uterus, as well as the mechanisms of cellular repair and regeneration.

## 4. Materials and Methods

### 4.1. Substances

The salts used for Locke Ringer’s solution were purchased from Vetec (Rio de Janeiro, Brazil), Nuclear (Porto Alegre, Brazil), and Dinâmica (Diadema, Brazil). The substances that were purchased included the following: isoprenaline, ibuprofen, nifedipine, eosin, hematoxylin, thiobarbituric acid, 1,1-diphenyl-2-picrylhydrazyl (DPPH), and 1,1,3,3-tetramethoxypropane (Merck, São Paulo, Brazil); formaldehyde and perchloric acid (Vetec, Rio de Janeiro, Brazil); diethylstilbestrol and prostaglandin F_2α_ (Cayman Chemical Company, Ann Arbor, MI, USA; oxytocin (União Química, São Paulo, Brazil); scopolamine and dipyrone (Neo Química Farmacêutica, Rio de Janeiro, Brazil); ketamine (Cristália Farmacêutica, Itapira, São Paulo, Brazil); and xylazine (Syntec Farmacêutica, Santana de Paranaíba, Brazil). Distilled water was used to dilute the substances and prepare the stock solutions, and diethylstilbestrol was dissolved in absolute alcohol (96° *v*/*v*). The carbogenic mixture used in the isolated organ bath was obtained from White Martins (Rio de Janeiro, Brazil). All substances were weighed using an analytical balance, the model GEHAKA AG 200 (São Paulo, Brazil).

### 4.2. Animals

Female Wistar rats (*Rattus norvegicus*), aged 8 weeks old (180–200 g), from the Animal Production Unit (UPA) of the Institute of Research in Drugs and Medicines (IPeFarM) of the Federal University of Paraíba (UFPB) were used. Virgin rats were kept under controlled environmental conditions of ventilation and temperature (21 ± 1 °C), with a 12 h light–dark cycle, with water ad libitum, and under dietary control with a balanced diet based on pellet-type food Nuvilab^®^ (Paraná, Brazil). All experimental procedures (UFPB Animal Use Ethics Committee: certificate No. 1886010520) were carried out following the guidelines for the ethical use of animals [51] and the National Council for the Control of Animal Experimentation (in Brazil) [52]. During the development of this study and the experimental protocols, there were no animal deaths.

### 4.3. Experimental Groups

Female Wistar rats were randomly divided into four experimental groups (*n* = 10 rats per group): (1) the control group (CG); (2) rats with primary dysmenorrhea (DysP); (3) rats with primary dysmenorrhea treated with scopolamine and dipyrone (DysP + Sco + Dip) (0.46/23 mg/kg); and (4) rats with primary dysmenorrhea treated with ibuprofen (DysP + IBU) (50 mg/kg).

### 4.4. In Vivo Approach: Dysmenorrhea Induction Protocol

#### 4.4.1. Induction of Primary Dysmenorrhea

**Figure 10:**
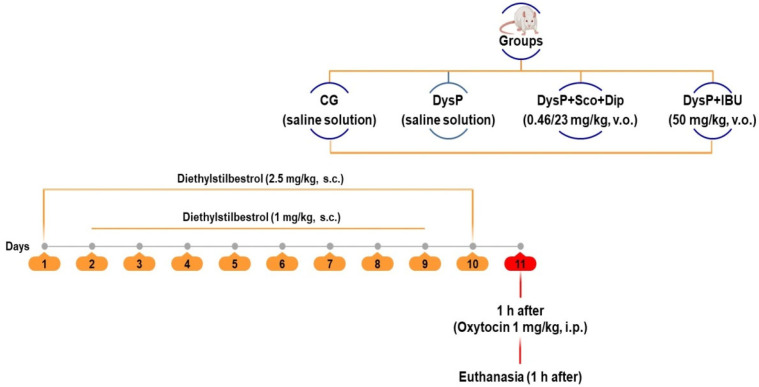
Primary dysmenorrhea induction protocol. CG = control group; DysP = primary dysmenorrhea group; DysP + Sco + Dip = primary dysmenorrhea + scopolamine with dipyrone (0.46/23 mg/kg) group; DysP + IBU = primary dysmenorrhea + ibuprofen (50 mg/kg) group. v.o.=orally; s.c. = subcutaneously; i.p. = intraperitoneal. Adapted from [53].

To induce dysmenorrhea, diethylstilbestrol (s.c.) was injected into the animals once a day for 10 consecutive days, on the 1st (first) and 10th (last) day (2.5 mg/kg) and with 1 mg/kg from the 2nd (second) to the 9th (ninth) day. After 24 h from the last administration, oxytocin (i.p.) (1 IU/kg) was injected into the animals [53] (Figure 10).

The animals in the control group were treated only with saline solution during all treatment days and 24 h before the experiment; 1 mg/kg of diethylstilbestrol was administered to standardize the animals’ estrous cycle. The animals treated with the standard drugs received them 1 h before the administration of oxytocin by the gavage method with scopolamine with dipyrone or ibuprofen, respectively. After the treatments, the female rats were euthanized by anesthesia (ketamine 100 mg/kg and xylazine 10 mg/kg, i.p.) and cervical dislocation, and the uterus and ovary were removed for later analyses [54,55]. Although both ketamine and xylazine may exert effects on different receptors and influence physiological responses, such as uterine or intestinal contractions, this does not imply that their use is inappropriate in all experimental circumstances. In the specific case of experiments involving removal of the uterus shortly after administration of anesthetics, the systemic effects of ketamine and xylazine on the animal’s body may be minimal or/and nullified, since the focus of the research is on the contractile reactivity of the isolated uterus and not on the general physiological responses of the animal. Therefore, the use of ketamine and xylazine may be appropriate in experimental models when the uterus is removed and analyzed outside the organism, in order to avoid interference with uterine function in vivo.

#### 4.4.2. Contortion Scoring

The scoring criteria for the behavioral experiments on the animals were producedby assigning scores to each writhing movement presented by the animals, in order to evaluate experimental primary dysmenorrhea. For this, the animals were placed in a glass box after the administration of oxytocin and were observed for 30 min by two evaluators, who underwent previous calibration training for behavioral evaluation. The scores assigned to each behavioral pattern are presented in Table 1 and were applied to calculate the writhing score, using the following formula described in the following equation [26,56].

Score = Σ (number of repetitions × scores)(1)

### 4.5. In Vitro Approach: Assessment of Uterine Reactivity

#### 4.5.1. Isolating the Uterus of Female Rats

The female rats were euthanized by anesthesia with ketamine (100 mg/kg) (i.p.) and xylazine (10 mg/kg) (i.p.); this was followed by a complementary method of cervical dislocation [57]. The abdominal cavity was opened with a longitudinal cut and the uterus was removed and cleaned of all connective and adipose tissue. Then, the uteri of female rats were stored in -80 freezers and/or liquid nitrogen for oxidative stress analyses and in 10% formalin for histological analyses. The uterine horns were separated in half, opened longitudinally, and suspended vertically by a cotton thread in bath tanks for isolated organs (6 mL) under a tension of 1 g and kept at rest for 40 min. The solution was renewed every 15 min [58]. After the stabilization period, a submaximal contraction was induced, recorded through isometric transducers coupled to a digital acquisition system, which was obtained with 60 mM KCl to verify the organ’s functionality.

Locke Ringer’s solution (adjusted to pH 7.4 with NaOH or 1N HCl) was carbonated with carbon and kept at 32 °C, and its composition (mM) was as follows: NaCl (154.0); KCl (5.6); CaCl_2_ (2.2); MgCl_2_ (2.1); glucose (5.6); and NaHCO_3_ (6.0).Locke Ringer’s solution was prepared and used only on the day of the experiment and then discarded, in order to rule out any instability in the pH of the solution over time.

#### 4.5.2. Effects of Primary Dysmenorrhea on Contractile Reactivity in the Uterus of Female Wistar Rats

After the stabilization period, two consecutive curves with cumulative concentrations of oxytocin, KCl, or PGF_2α_ were obtained. The results were evaluated by comparing the amplitude of the contractile response of the uterus of the dysmenorrhea group with that obtained by the average of the maximum amplitudes of the control curves [59].

#### 4.5.3. Effects of Primary Dysmenorrhea on Relaxant Reactivity in the Uterus of Female Wistar Rats

In utero contraction was induced using oxytocin (10−2 IU/mL) or KCl (60 mM). After the formation of the tonic component, isoprenaline or nifedipine was added cumulatively to the organ bath of all groups in different preparations [59]. Comparisons were made between the dysmenorrhea groups, with the means of the maximum amplitudes of the control curves, based on the Emax and pEC_50_ values of the relaxant substances.

### 4.6. Evaluation of Histopathological Changes in the Uterus Induced by Primary Dysmenorrhea

The uterus samples from each female rat were isolated and fixed in 10% buffered formaldehyde solution and embedded in paraffin. Subsequently, they were cut with a microtome (4 μm thick), mounted on histological slides, and deparaffinized in xylene for 30 min. They were then hydrated in absolute alcohol in decreasing concentrations for 25 min and washed in running water for 5 min and then washed in distilled water. The samples were treated with Harris hematoxylin for 1 min, washed again in distilled water for 5 min, and stained again with eosin for 3 min and were then also washed in running water for another 30 s. The slides were dehydrated in increasing concentrations of alcohol, diaphanized in xylene, and mounted with Entellan™ (Merck KGaA, Darmstadt, Germany).

The following parameters were analyzed in the uterine sections: the thickness of the uterine layers (endometrium and myometrium) and the number of uterine glands and blood vessels. Photomicrographs of the slides were captured using a camera attached to an optical microscope (Alphatec^®^, Lima, Peru). The methodology used in the microscopic imaging, image segmentation, and definition of morphometric conditions was the method described by Caliari [60]. Ten images and 5 sections from each animal were obtained for tissue cutting. The best images were selected according to the technique/method used to proceed with the analysis and conclusions of the results.

### 4.7. Evaluation of the Effects of Primary Dysmenorrhea on the Balance Between Oxidative Stress and Antioxidant Defenses in Female Wistar Rats

#### Analysis of Malondialdehyde (MDA) Levels and Total Antioxidant Capacity (TAC) in Uterine Tissue

The homogenized uterine horns were frozen at −20 °C. The tissue was then weighed, macerated, and homogenized with 10% KClat a 1:1 ratio. Subsequently, the samples were centrifuged (1198× *g*/10 min) and the supernatant was separated for testing.

Lipid peroxidation assessment was performed by quantifying MDA production using the thiobarbituric acid reactive species (TBARS) quantification method. After obtaining the uterine horn homogeneity, 250 µL aliquots were incubated at 37 °C in a water bath for 60 min. Subsequently, the samples were precipitated with 400 µL of 35% perchloric acid and centrifuged at 16,851× *g* for 20 min at 4 °C. The supernatant was transferred to Eppendorf^®^ tubes; 400 µL of 0.6% thiobarbituric acid (TBA) was added to the samples and incubated at 95–100 °C for 1 h. After cooling, the samples were read using a spectrophotometer at 532 nm [61].

To evaluate the total antioxidant capacity (TAC) of the uterine horns, the colorimetric DPPH reduction method was used. It was based on the sample’s ability to reduce the DPPH radical, which had a purple coloration, to 1,1-diphenyl-2-picryl hydrazine, which had a transparent color, detected using a spectrophotometer. Thus, 50 µL of plasma or uterine homogeneity and 2 mL of DPPH solution dissolved in absolute ethanol (0.012 g/L) were added to a centrifuge tube and protected from light; then, the tubes were vortexed for 10 s and kept at rest for 30 min. Then, the samples were centrifuged at 7489× *g* for 15 min at 20 °C. The supernatant was read using a spectrophotometer at 515 nm (Infitek, Shanghai, China) [62]. Analyses were performed to compare the levels of MDA (μmol/L sample) or TAC (%) between the CG and the DysP, DysP + Sco + Dip, and DysP + IBU groups.

### 4.8. Statistical Analysis

The contractile reactivity of the rat uteri was evaluated by determining the maximum effect (E_max_) and the negative logarithm to the base ten of the concentration of a substance that produced 50% of the maximum effect (pEC_50_) of oxytocin, KCl, or PGF_2α_ for animals with primary dysmenorrhea and compared to the control group. Relaxant reactivity was analyzed as the reverse percentage of the contraction induced using oxytocin or KCl and the results were evaluated by comparing the amplitude of the relaxant response of the uterus of female rats in the primary dysmenorrhea group with that obtained by the mean of the maximum amplitudes of the control curves.

All data were expressed as the mean ± standard error of the mean (s.e.m.) and statistically analyzed using the “t” test (unpaired) or one-way analysis of variance (ANOVA) followed by Tukey’s post-test, with differences between means considered significant when *p* < 0.05. The pEC_50_ was calculated by nonlinear regression for all experiments performed. Every possible comparison between the study groups was considered. All results were analyzed by the program Graph Pad Prism^®^ 5.01 (Graph Pad Software Inc., San Diego, CA, USA).

## 5. Conclusions

Thus, the experimental model of primary dysmenorrhea that was the object of this study was validated by promoting in vivo clinical signs of the disease, which were attenuated by standard drugs used in clinical practice, as well as promoting uterine hypercontractility and increased oxidative stress in the in vitro studies, which are aspects involved in the pathophysiology of DysP, suggesting that this study was able to replicate the DysP model, validating the implementation and standardization of the method. In summary, we established and standardized a model of primary dysmenorrhea in virgin female rats, whose characteristics were preserved throughout the estrous cycles of these rodents. This model may provide an excellent approach for screening new drugs aimed at treatment and/or prevention, as well as exploring the pathophysiological mechanisms of DysP.

## Figures and Tables

**Table 1:** Scores for animal behavioral patterns. A scoring table for the behavioral pattern presented by the animals. (0) Animals with normal posture. (1) Movements in the oblique side of the body. (2) Dorsiflexion movement in the hind leg or the extension of the body with frequent pelvic rotation. (3) The contraction of the abdominal muscle or extension of the hind limbs [25,26].

Scores	Features
0	Normal posture
1	Oblique side of body
2	Dorsiflexion of hind leg
2	Body extension with frequent pelvic lateral rotation
3	Abdominal muscle contraction
3	Extension of hind limbs

## Data Availability

Data are unavailable due to privacy.

## Abbreviations

[Ca^2+^]_c_	Cytosolic calcium concentration
ACh	Acetylcholine
COX2	Cyclooxygenases type 2
COXs	Cyclooxygenases
DPPH	1,1-diphenyl-2-picrylhydrazyl radical
DysP	Primary dysmenorrhea
EDTA	Ethylenediamine tetra-acetic acid
E_max_	Maximum effect
i.p	Intraperitoneal
DysP + IBU	Primary dysmenorrhea group treated with ibuprofen
IPeFarM	Institute of Research in Drugs and Medicines
M_3_	Receptor muscarinic type 3
MDA	Malondialdehyde
OT	Oxytocin receptor
pEC_50_	The negative logarithm to the base ten of the concentration of a substance that produced 50% of its maximum effect
PGF_2α_	Prostaglandin F_2α_
PG’s	Prostanoids
PLA_2_	Phospholipase A_2_
ROS	Free radicals
s.c.	Subcutaneous
s.e.m.	Standard error of the mean
TAC	Total antioxidant capacity
TBA	Thiobarbituric acid
TBARS	Thiobarbituric acid reactive species
UFPB	Federal University of Paraíba
